# Arterial stiffness and ankle brachial index in patients with rheumatoid arthritis and inflammatory bowel disease

**DOI:** 10.1186/s12950-016-0110-y

**Published:** 2016-01-08

**Authors:** Kadir Ozturk

**Affiliations:** Department of Gastroenterology, Gulhane School of Medicine, Ankara, Turkey

## To the Editor;

We read the article “Comparison of inflammation, arterial stiffness and traditional cardiovascular risk factors between rheumatoid arthritis and inflammatory bowel disease” by Fan et al. [1] with great interest. In this very-well designed study, authors found that pulse wave velocity (PWV) levels were similar between rheumatoid arthritis (RA), inflammatory bowel disease (IBD) and control groups. In addition, they also showed that ankle brachial ındex (ABI) levels were higher in controls than IBD, but not RA. They concluded that traditional risk factors, but not inflammatory markers, are major parameters asscociated with arterial stiffness. These findings provide new information about the asscociation between inflammatory diseases and atherosclerosis.

In this study, although RA patients have higher Framingham risk score and number of hypertensive patients, there was no significant difference between RA and control groups in PWV and ABI levels. Arterial stiffness has been known as an independent predictor cardiovascular mortality and is increased in rheumathologic diseases despite a low risk for cardiovascular disease according to Framingham score [2]. Moreover, in IBD patients, ABI levels were significantly decreased when compared to control subjects, but PWV levels were not correlated with ABI. Whereas a negative correlation between PWV and ABI was demonstrated in individuals without organic heart disease [3].

We suggest some possible explanations for the lack of increased PWV and decreased ABI in patients with RA and IBD than controls. In present study, authors did not examine renal function tests and hemoglobin levels. We know that patients with chronic renal failure and anemia have increased risk for cardiovascular events and peripheral artery disease [4, 5]. Moreover, they did not examine the association of disease extent and duration with vascular parameters. We think that as disease extent and duration increased, risk of atherosclerosis will be increased due to long-term exposure to inflammation.

Before making certain comments on these findings, we think that this study should be reconsidered in light of the above mentioned suggestions. This could provide the readers of the journal clearer information regarding the effects of inflammatory diseases in atherosclerosis.

Authors’ Response:

Dear Editors of Journal of Inflammation,

We are writing in response to the letter concerning our manuscript. We appreciate the interest in our work. As noted, we did not observe a significant increase in mean baPWV in RA patients [1]. Whilst RA has been associated with an increased prevalence of arterial stiffness. there are other other studies that also failed to find an increase in PWV [6, 7] in these patients. Another study reported that only about 30 % of RA patients had increased arterial stiffness [8]. In IBD, PWV has been found to be increased [9] or unchanged [10]. Thus, findings on arterial stiffness in RA and IBD patients have varied between studies, possibly due to the difference in demographics of patients studied.

In our study, most patients were well controlled by treatment with disease modifiying drugs. It is known that disease modifying drugs improve endothelial dysfunction and reduce arterial stiffness [11–13]. Disease duration and activity are also likely to influence arterial stiffness [8, 14]. We thus examined the correlations between disease duration and vascular parameters. We did not examine correlations with disease activity since most patients were in remission. PWV was correlated with disease duration in patients with IBD (r = 0.343, *p* = 0.026) but not with RA. Other investigators also did not find correlations between PWV and disease duration or activity [15]. This study also reported that among the many factors related to arterial stiffness, only old age and high systolic blood pressure were major determinants, which is concistent with our findings. ABI was not corrrelated with vascular parameters in either RA or IBD. Although negative correlation between PWV and ABI have been demonstrated in individuals without organic heart disease [3], we did not observe such inverse correlation in our study.

Finally, we appreciate and agree with the comment that patients with chronic renal failure and anaemia have increased risk for cardiovascular events and peripheral artery disease [4, 5]. As suggested we have examined data on renal function (urea, creatinine) and haemoglobin, which are all within the normal range (Table 1).

**Table 1:** Laboratory results of study population

	RA (*n* = 43)	IBD (*n* = 42)
Urea, mg/dL	5.6 ± 2.2	5.8 ± 2.5
Creatinine, μmoL/L	68.7 ± 16.2	79.9 ± 38.1
Hemoglobin, g/L	134.1 ± 18.4	137.4 ± 16.5

## References

[1] Fan F, Galvin A, Fang L, White DA, Moore XL, Sparrow M (2014). Comparison of inflammation, arterial stiffness and traditional cardiovascular risk factors between rheumatoid arthritis and inflammatory bowel disease. J Inflamm (Lond).

[2] Sacre K, Escoubet B, Pasquet B, Chauveheid MP, Zennaro MC, Tubach F (2014). Increased arterial stiffness in systemic lupus erythematosus (SLE) patients at low risk for cardiovascular disease: a cross-sectional controlled study. PLoS One.

[3] Su HM, Lee KT, Chu CS, Lee MY, Lin TH, Voon WC (2007). Effects of heart rate on brachial-ankle pulse wave velocity and ankle-brachial pressure index in patients without significant organic heart disease. Angiology.

[4] Ford ML, Tomlinson LA, Chapman TP, Rajkumar C, Holt SG (2010). Aortic stiffness is independently associated with rate of renal function decline in chronic kidney disease stages 3 and 4. Hypertension.

[5] Foster MC, Ghuman N, Hwang SJ, Murabito JM, Fox CS (2013). Low ankle-brachial index and the development of rapid estimated GFR decline and CKD. Am J Kidney Dis.

[6] Cypiene A, Laucevicius A, Venalis A, Ryliskyte L, Dadoniene J, Petrulioniene Z (2007). Non-invasive assessment of arterial stiffness indices by applanation tonometry and pulse wave analysis in patients with rheumatoid arthritis treated with TNF-alpha blocker remicade (infliximab). Proc West Pharmacol Soc.

[7] Scolnik M, Saucedo C, Navarta OD, Ferreyra GL, Lancioni E, Varela GF (2012). Lipid alterations and measurement of arterial stiffness in rheumatoid arthritis. Arthritis Rheum.

[8] Sliem H, Nasr G (2010). Change of the aortic elasticity in rheumatoid arthritis: relationship to associated cardiovascular risk factors. Journal of cardiovascular disease research.

[9] Zanoli L, Cannavo M, Rastelli S, Di Pino L, Monte I, Di Gangi M (2012). Arterial stiffness is increased in patients with inflammatory bowel disease. J Hypertens.

[10] Theocharidou E, Mavroudi M, Soufleris K, Griva T, Giouleme O, Athyros VG (2013). Aortic stiffness in patients with inflammatory bowel diseases. Hellenic J of Atherosclerosis.

[11] Maki-Petaja KM, Elkhawad M, Cheriyan J, Joshi FR, Ostor AJ, Hall FC (2012). Anti-tumor necrosis factor-alpha therapy reduces aortic inflammation and stiffness in patients with rheumatoid arthritis. Circulation.

[12] Protogerou AD, Zampeli E, Fragiadaki K, Stamatelopoulos K, Papamichael C, Sfikakis PP (2011). A pilot study of endothelial dysfunction and aortic stiffness after interleukin-6 receptor inhibition in rheumatoid arthritis. Atherosclerosis.

[13] Tam LS, Shang Q, Li EK, Wang S, Li RJ, Lee KL (2012). Infliximab is associated with improvement in arterial stiffness in patients with early rheumatoid arthritis -- a randomized trial. The Journal of rheumatology.

[14] Lee JH, Cho KI, Kim SM (2012). Carotid arterial stiffness in patients with rheumatoid arthritis assessed by speckle tracking strain imaging: its association with carotid atherosclerosis. Clin Exp Rheumatol.

[15] Kim YS, Sung YK, Choi CB, Uhm WS, Kim TH, Shin JH (2012). The major determinants of arterial stiffness in Korean patients with rheumatoid arthritis are age and systolic blood pressure, not disease-related factors. Rheumatol Int.

